# Pulmonary Hypertension and Survival among Non-Small Cell Lung Cancer Patients: A Retrospective Cohort Study in the U.S. Military Health System

**DOI:** 10.3390/jcm13113217

**Published:** 2024-05-30

**Authors:** Joel A. Nations, Jie Lin, Amie B. Park, Craig D. Shriver, Kangmin Zhu

**Affiliations:** 1 Veterans Affairs Medical Center, Washington, DC 20422, USA; 2 Department of Medicine, Uniformed Services University, Bethesda, MD 20814, USA; 3 John P. Murtha Cancer Center Research Program, Department of Surgery, Uniformed Services University of the Health Sciences, Bethesda, MD 20817, USAcraig.shriver@usuhs.edu (C.D.S.); kangmin.zhu@usuhs.edu (K.Z.); 4 Henry M. Jackson Foundation for the Advancement of Military Medicine, Bethesda, MD 20817, USA; 5 Department of Preventive Medicine and Biostatistics, Uniformed Services University of the Health Sciences, Bethesda, MD 20814, USA; 6 Department of Surgery, Walter Reed National Military Medical Center, Bethesda, MD 20814, USA

**Keywords:** lung cancer, mortality, pulmonary hypertension

## Abstract

**Background:** Lung cancer is one of the most lethal cancers with survival being closely related to stage and influenced by comorbid illness. The survival implications of pulmonary hypertension (PH) on patients with non-small cell lung cancer (NSCLC) have only been evaluated in small cohorts, with limited long-term follow-up. **Methods:** We conducted a retrospective cohort study of 7946 patients with NSCLC diagnosed in the MHS. This study evaluated the survival impact of PH in patients diagnosed with NSCLC in the MHS. Patients were classified as having and not having PH. We stratified PH into those diagnosed before the diagnosis of NSCLC and those diagnosed after NSCLC diagnosis. **Results:** Relative to patients without PH, patients with PH diagnosed before NSCLC had an increased risk of death (HR = 1.15 [95% CI, 1.02–1.29]). The increased risk of death was more obvious for patients with PH diagnosed after NSCLC compared with those without PH (HR = 2.74 [95% CI, 2.51–2.99]). The results were similar when stratified by patient demographics. **Conclusions**: In the MHS, PH is associated with worsened NSCLC survival, regardless of when it is diagnosed. When PH is diagnosed after NSCLC, it is associated with a marked reduction in survival, and this finding may suggest a potential role for monitoring pulmonary pressures in NSCLC patients. Furthermore, as specific PH therapy exists, some NSCLC patients with PH may be candidates for therapy.

## 1. Introduction

Lung cancer is the second most common cancer in males and females but continues to be the most lethal, causing an estimated 22% of cancer deaths in the US [1]. Stage at diagnosis remains the primary determinant of survival and comorbid conditions have also been linked to survival [2,3,4]. In many diseases affecting the human lung, pulmonary hypertension (PH) when present, portends a poor prognosis. PH in patients with sarcoidosis [5], idiopathic pulmonary fibrosis [6], and COPD [7] is associated with shortened survival. Little is known, however, about the impact of PH on the survival of patients with non-small cell lung cancer (NSCLC). Several small studies have evaluated the risks of abnormal pulmonary hemodynamics on NSCLC outcomes, focused mainly on the physiologic assessment of patients being considered for NSCLC resection [8,9,10,11]. Other studies have evaluated non-invasive surrogate markers of PH [12]. The results of these studies have shown mixed results. In most studies on NSCLC resection outcomes, pre-existing PH has not been shown to lead to postoperative morbidity or mortality. Conversely, non-invasive markers of PH in lung cancer patients, specifically enlargement of the pulmonary artery on CT imaging, have been shown to predict decreased survival [13]. To date, these studies were usually small, had limited follow-up, and showed inconsistent results. Furthermore, these studies used surrogate markers of PH rather than ICD diagnostic coding and were limited to single institutions. To further investigate the relationship between PH and NSCLC outcomes this study aimed to evaluate the long-term survival impact of comorbid PH ascertained by ICD coding in NSCLC patients diagnosed or treated at all military treatment facilities.

## 2. Materials and Methods

### 2.1. Data Source

This study was based on the MilCanEpi data system, which links databases from the Department of Defense (DoD)’s Central Cancer Registry (CCR) and the Military Health System (MHS) Data Repository (MDR). The linked database has been described previously [14]. Briefly, the CCR contains data on demographics, cancer diagnosis, tumor characteristics, cancer treatment, vital status, and other information on Military Health System (MHS) beneficiaries with cancer diagnosed and/or treated at military treatment facilities (MTFs). The MDR contains administrative and medical claims data from in-patient and out-patient services provided directly at MTFs or services provided at civilian medical facilities and paid by DoD. The MDR database includes information on diagnoses of medical conditions and treatment procedures. The diagnoses and procedures were coded with the International Classification of Disease (ICD) or Current Procedural Terminology (CPT) codes. At the time of data acquisition, the linked database contains data for cancer patients diagnosed between 1998 and 2014. The data linkage and the use of MilCanEpi for research were approved by the institutional review board of the Uniformed Services University.

### 2.2. Study Population

Patients included in this study were those 18 or older with microscopically confirmed primary malignant NSCLC diagnosed between 1998 and 2014. Patients with diagnosis from death certificate or autopsy only were excluded. Cancer site and histology were classified using the topography (C34.0 to C34.3, C34.8, C34.9) and morphology codes (8050–8078, 8083, 8084, 8250–8260, 8480–8490, 8570–8574, 8140, 8211, 8230, 8231, 8323, 8550, 8551, 8576, 8010–8012, 8014–8031, 8035, 8310) of the International Classification of Disease for Oncology, 3rd edition (ICD-O-3). We excluded patients with multiple primary cancers from the study to minimize the potential effects of other cancers on death.

### 2.3. Study Variables

The outcome of this study was all-cause death. All-cause death was used because the data on cancer-specific death were incomplete. The variable of study was diagnosis of pulmonary hypertension. PH diagnosis was identified from the MDR inpatient and outpatient records using the International Classification of Disease, 9th edition (ICD-9) codes (416, 4160, 4161, 4162, 41519, 4168, 4169) and the International Classification of Disease, 10th edition (ICD-10) codes (I27, I270, I271, I272, I2720, I2721, I2722, I2723, I2724, I2729, I2781, I2782, I2789, I279, I2790). Time at diagnosis of PH was determined based on the first in-patient or outpatient record in the data. PH occurring both before and after NSCLC diagnosis was identified. Patients were classified as having PH before NSCLC if they had ever had the diagnosis before NSCLC (despite whether there was a PH diagnosis after NSCLC). Patients were classified as having PH after NSCLC if they had the diagnosis solely after NSCLC. The group of no PH were patients who never had PH in their medical records.

Tumor stages were defined according to American Joint Committee on Cancer’s (AJCC) TNM system criteria, the 6th edition, including stages I, II, III, and IV [15]. Site-specific cancer-directed surgery codes were used to define cancer-directed surgery types and further grouped into “surgery received”, “surgery not received”, and “unknown if surgery was received”. Chemotherapy was grouped into “chemotherapy therapy received”, “chemotherapy therapy not received”, or “unknown if chemotherapy was received”. Receipt of radiation therapy was grouped into similar three groups.

### 2.4. Statistical Analysis

Differences in patient characteristics by PH status were first compared using chi-square test. We then performed survival analysis. Patients were followed to death or the study end date, 31 December 2015. If a patient died during the follow-up, the survival time was calculated as the time from diagnosis to death. For patients who did not die during the study period, their survival time was censored on the study ending date. The relationship between PH and all-cause survival was subsequently analyzed by time at diagnosis of PH. When PH was diagnosed before NSCLC was analyzed, we used conventional Cox regression model. When PH diagnosed after NSCLC was the variable of analysis, we used time-dependent variable for PH to control for the potential impact of immortal time bias [16].

Hazard ratios (HRs) and 95% confidence interval [95% CI] were estimated, adjusting for continuous age, sex (male, female), race (White, Black, Asian/Pacific Islander, Other/Unknown), year of diagnosis, tumor stage (stages I, II, III and IV), surgery (yes, no, unknown), chemotherapy (yes, no, unknown), and radiation therapy (yes, no, unknown). Statistical Analysis System (SAS) software, Version 9.3 for Windows (SAS Institute, Inc., Cary, NC, USA) was used to perform statistical analyses. All tests of significance were two-tailed and performed at an alpha of 0.05.

## 3. Results

The study population included 7946 patients, of which 8% were <50 years old, 39% were 50–64 years old and 54% were 65 years or older. Males predominated (64%) as did White race (77%). Approximately, 33% had early-stage (stage I and stage II) NSCLC. Therapy included chemotherapy (55%) radiation therapy (54%) and 65% had NSCLC-related surgery (Table 1). Median follow-up was 353 days (25% quartile: 142 days; 75% quartile: 684 days).

While PH was not identified in 6459 (81%) of NSCLC patients, it was diagnosed in 1487 (19%) of patients, with 6% having PH diagnosed before NSCLC and 13% having PH diagnosed after NSCLC diagnosis (Table 1). Patients with PH diagnosed before NSCLC were younger than those without PH and patients with PH diagnosed after NSCLC were more likely to be women. Both groups with PH were more likely to be White and diagnosed in more recent years than the group without PH. Additionally, both PH groups were more likely to have stage 1 tumors and less likely to have stage 4 tumors at diagnosis, compared to patients without PH. Patients with baseline PH were less likely to receive surgical therapy compared to patients without PH (26% versus 33%, *p* = 0.003), whereas patients with PH diagnosed after NSCLC diagnosis were more likely to have surgery than those without PH (44.5% versus 33.4%, *p* < 0.001). The same tendencies were observed for chemotherapy. On the contrary, patients without PH were more likely to have radiation therapy, or unknown status in the therapy, than the PH groups.

Table 2 shows the relationship between PH diagnosed before NSCLC and survival. PH diagnosed before NSCLC was associated with increased death relative to patients with PH (HR 1.15 and 95% CI = 1.02–1.29), which was observed in most subgroups stratified by age, sex, race, or tumor stage. The association between PH and NSCLC was stronger for PH diagnosed after PH (HR 2.74 and 95% CI = 2.51–2.99), relative to patients without PH (Table 3). This association was observed across subgroups stratified by age, sex, race, and tumor stage.

## 4. Discussion

In the current study, which represents the largest study to date investigating the association between PH and long-term NSCLC outcomes, we identified a clear association between a diagnosis of PH and reduced all-cause survival in patients with NSCLC. There appears to be a much stronger association between PH diagnosed after an NSCLC diagnosis and long-term survival, as opposed to the more modest apparent association between baseline PH and NSCLC survival. Our findings agree with Yang et al., who examined lung cancer survival based on a pre-treatment echocardiographic assessment for PH in 612 patients with NSCLC [17]. The authors reported that three- and five-year overall survival were both significantly lower in patients with baseline PH, although they excluded patients with baseline COPD, left heart disease, and other conditions. Also in agreement with our study is the research of Eul and colleagues who reported a strong association between the non-invasive surrogate for PH, pulmonary arterial enlargement on CT imaging, and both overall and disease-free survival in 670 patients with NSCLC [13]. While our results confirmed the findings of these previous studies in a cohort of nearly 8000 NSCLC patients following a median of 353 days, we demonstrated that PH diagnosed both before and after NSCLC increased the risk of all-cause death.

Although the features of our data limit a granular determination of the etiology of PH in these NSCLC patients, one can reasonably postulate three general mechanisms. The first would be the presence of pre-existing cardiopulmonary conditions and associated PH. Second, PH may also have been diagnosed echocardiographically during preoperative evaluation or for baseline assessment prior to the initiation of potentially cardiotoxic chemotherapy. Another potential mechanism may be that PH subsequent to NSCLC diagnosis is potentially a complication from lung cancer therapy.

The relationship between NSCLC and PH is complex. First, NSCLC and PH may share the same risk factors. For instance, smoking increases lung cancer risk but is also a primary risk for the development of COPD and CHF, for which coexistent PH is well described [18,19]. There is also an increased risk for both NSCLC and PH in diffuse lung diseases such as IPF [20] and sarcoidosis [21] and when present, PH portends a poor prognosis for patients with diffuse parenchymal lung diseases. Second, PH may result from therapies of NSCLC. NSCLC lung resection has been associated with the development of postoperative PH with an associated worsened survival [22,23]. PH is also recognized as a potential effect of systemic treatment with various chemotherapy agents [24], and radiotherapy for lung cancer [25]. However, these treatments were adjusted in our multivariable analyses, suggesting that PH may have independent effects on survival. Third, PH may be directly related to NSCLC. There is emerging evidence that PH may develop in NSCLC as the result of vascular remodeling, perivascular inflammatory cell accumulation, or through a phenomenon termed pulmonary tumor thrombotic microangiopathy [26]. Thus, the association of PH with NSCLC may be associated with shared risk factors, comorbid illnesses that cause PH, or diagnosis of lung cancer or its therapies that directly lead to PH.

The diagnosis of PH in NSCLC is not rare. For instance, Eul and colleagues found that 22.5% of NSCLC patients had a PA/A ratio > 1, a surrogate marker for PH. Using echocardiographic estimates of PH, Yang reported that 20% of NSCLC patients had PH. Likewise, 19% of NSCLC patients in our study were diagnosed with PH. While 6% of NSCLC patients in our study had baseline PH, this diagnosis was associated with a lower likelihood of resection and likely reflects PH as a marker for increased comorbid disease burden, which may affect a patient’s candidacy for therapy.

It is intuitive that PH in patients with NSCLC would be associated with worsened survival and recent research has shown this to be true. However, recommendations for the physiologic evaluation of patients under consideration for NSCLC resection do not currently include evaluation for PH [27], and screening for PH in NSCLC patients is not recommended. As therapy for secondary PH is often not indicated, NSCLC-associated PH may not provide a target for specific therapy. One clinical implication of this research is to better understand the occurrence of PH in NSCLC patients. Another clinical implication is the consideration of screening NSCLC patients for PH, which may be particularly relevant to NSCLC patients undergoing lung resection. Lastly, these findings should generate further research to better understand the mechanistic interplay between PH and NSCLC and to ascertain whether PH in NSCLC patients may offer a target for therapy.

This study has limitations. First, we used a single outpatient or inpatient record to define PH. As a result, there is a possibility that some PH defined based on a single outpatient record might not be a confirmed diagnosis and thus the PH groups might include some patients without PH. Nevertheless, this potential misclassification might have only diluted the association between PH and survival. Second, in this study we stratified patients with PH diagnosed before NSCLC and those with PH diagnosed after NSCLC. Patients diagnosed with PH after their lung cancer diagnosis may simply have had PH existent but undiagnosed before NSCLC diagnosis or diagnosed before the period of the data. Therefore, it is possible that the true difference between pre-diagnosis PH and post-diagnosis PH in its association with survival might be smaller than the observed one. Third, although we controlled for multiple potential confounding factors in this study, we did not control for the presence or absence of COPD, cardiac disease, or interstitial lung disease, as well as other factors such as genetics, infections, previous medication use, which are associated with PH, because of the unavailability of the information or uncertain temporal relationships between these factors and PH (due to the potential data issues described above). Thus, we do not exclude the possibility of residential confounding by these factors. Fourth, in this retrospective cohort study, we found a correlation between increased risk of death in patients with NSCLC and PH, which does not prove causality. Lastly, all patients were cared for in the U.S. Military Health System between 1998 and 2014, and whether these results can be extrapolated to other populations is unclear.

## 5. Conclusions

Although there has been recent progress, non-small cell lung cancer remains a lethal cancer. To date, research to understand the survival impact of pulmonary hypertension in non-small cell lung cancer patients has been limited. Our findings, the first to evaluate the long-term survival impact of pulmonary hypertension in non-small cell lung cancer patients, show a correlation between an increased risk of death for patients with non-small cell lung cancer and pulmonary hypertension. While not a surprising finding, further research is warranted to understand the mechanisms for the development of pulmonary hypertension in non-small cell lung cancer. Furthermore, this may provide a potential beneficial role for pulmonary hypertension screening in patients with non-small cell lung cancer and during follow-up after therapy. Lastly, pulmonary hypertension in patients with non-small cell lung cancer may provide a target for therapy.

## Figures and Tables

**Table 1:** Characteristics of non-small cell lung cancer (LC) patients by pulmonary hypertension status in the U.S. Military Health System, 1998–2014.

Variable	No PH (N = 6459)	PH after LC ^1^ (N = 1036)	PH before LC ^2^ (N = 451)	*p*-Value (PH after vs. No PH)	*p*-Value (PH before vs. No PH)
**Age**				0.131	<0.001
<50	520 (8.05)	65 (6.27)	14 (2.22)		
50–64	2560 (39.63)	412 (39.77)	109 (24.17)		
65 or older	3379 (52.31)	559 (53.96)	328 (72.73)		
**Sex**				0.021	0.421
Male	4174 (64.62)	631 (60.69)	283 (62.75)		
Female	2285 (35.38)	405 (39.09)	168 (37.25)		
**Race**				<0.001	0.005
White	4903 (75.91)	822 (79.34)	376 (83.37)		
Black	780 (12.08)	136 (13.13)	38 (8.43)		
Asian/Pacific Islander	553 (8.56)	61 (5.89)	27 (5.99)		
Other/Unknown	223 (3.45)	17 (1.64)	10 (2.22)		
**Diagnosis Year**				<0.001	<0.001
1998–1999	1122 (17.37)	130 (12.55)	40 (8.87)		
2000–2004	2262 (35.02)	390 (37.64)	101 (22.39)		
2005–2009	1654 (25.61)	315 (30.41)	146 (32.37)		
2010–2014	1421 (22.00)	201 (19.40)	164 (36.36)		
**Tumor stage**				<0.001	0.008
Stage I	1580 (24.46)	349 (33.69)	126 (27.94)		
Stage II	440 (6.81)	87 (8.40)	16 (3.55)		
Stage III	1620 (25.08)	263 (25.39)	132 (29.27)		
Stage IV	2509 (38.85)	307 (29.63)	157 (34.81)		
Unknown stage	310 (4.80)	30 (2.90)	20 (4.43)		
**Surgery**				<0.001	0.003
No	4239 (65.63)	568 (54.83)	332 (73.61)		
Yes	2159 (33.43)	461 (44.50)	116 (25.72)		
Unknown	61 (0.94)	7 (0.68)	3 (0.67)		
**Chemotherapy**				<0.001	<0.001
No	3536 (54.75)	508 (49.03)	295 (65.41)		
Yes	2735 (42.34)	505 (48.75)	144 (31.93)		
Unknown	188 (2.91)	23 (2.22)	12 (2.66)		
**Radiation therapy**				0.017	<0.001
No	3438 (53.23)	591 (57.05)	283 (62.75)		
Yes	2748 (42.55)	416 (40.15)	162 (35.92)		
Unknown	273 (4.23)	29 (2.80)	6 (1.33)		

PH, pulmonary hypertension; LC, lung cancer. ^1^ PH after = patients with a PH diagnosis occurred solely after LC diagnosis (N = 1036). ^2^ PH before = patients with a PH diagnosis ever occurred before LC diagnosis, including patients with PH diagnosed solely before LC (N = 316), and those with PH diagnosed both before and after LC (N = 135).

**Table 2:** Overall and stratified hazard ratios of all-cause death for pulmonary hypertension (PH) diagnosed before lung cancer (including before only and both before and after) among patients diagnosed from 1998 to 2014 in the U.S. Military Health System.

Variables	Numbers	Adjusted HR ^1^ (95% CI)
	All Cases/Deaths	
Overall		
No	6459/4404	1.00 (ref.)
Yes	451/310	1.15 (1.02–1.29)
By Age		
<50		
No	520/271	1.00 (ref.)
Yes	14/6	3.77 (1.60–8.86)
50–64		
No	2560/1644	1.00 (ref.)
Yes	109/64	1.02 (0.79–1.31)
65 or older		
No	3379/2489	1.00 (ref.)
Yes	328/240	1.17 (1.02–1.33)
Sex		
Male		
No	4174/2995	1.00 (ref.)
Yes	283/205	1.06 (0.92–1.23)
Female		
No	2285/1409	1.00 (ref.)
Yes	168/105	1.37 (1.12–1.68)
By Race		
White		
No	4903/3432	1.00 (ref.)
Yes	376/256	1.05 (0.92–1.20)
Black		
No	780/518	1.00 (ref.)
Yes	38/29	2.03 (1.37–2.99)
Asian/Pacific Islander		
No	553/329	1.00 (ref.)
Yes	27/18	1.55 (0.95–2.52)
Other		
No	223/125	1.00 (ref.)
Yes	10/7	2.62 (1.14–6.04)
By tumor stage		
Stage I		
No	1580/553	1.00 (ref.)
Yes	126/53	1.41 (1.06–1.89)
Stage II		
No	440/246	1.00 (ref.)
Yes	16/8	0.93 (0.45–1.94)
Stage III		
No	1620/1238	1.00 (ref.)
Yes	132/100	1.33 (0.92–1.40)
Stage IV		
No	2509/2181	1.00 (ref.)
Yes	157/133	1.09 (0.91–1.30)
Unknown stage		
No	310/186	1.00 (ref.)
Yes	20/16	1.57 (0.93–2.68)

^1^ HRs were estimated from multivariable Cox proportional hazard models. HRs were adjusted for age (as continuous variable), sex, race, year of diagnosis, tumor stage, surgery, chemotherapy, and radiation except the stratified variable itself. HR = hazard ratio; CI = confidence interval.

**Table 3:** Overall and stratified hazard ratios of all-cause death for pulmonary hypertension (PH) after lung cancer diagnosis among patients diagnosed from 1998 to 2014 in the U.S. Military Health System.

Variables	Numbers	Adjusted HR ^1^ (95% CI)
	All Cases/Deaths	
Overall		
No	6459/4404	1.00 (ref.)
Yes	1036/606	2.74 (2.51–2.99)
By Age		
<50		
No	520/271	1.00 (ref.)
Yes	65/43	2.33 (1.66–3.27)
50–64		
No	2560/1644	1.00 (ref.)
Yes	412/236	2.76 (2.40–3.18)
65 or older		
No	3379/2489	1.00 (ref.)
Yes	559/327	2.78 (2.47–3.13)
Sex		
Male		
No	4174/2995	1.00 (ref.)
Yes	631/384	2.61 (2.34–2.91)
Female		
No	2285/1409	1.00 (ref.)
Yes	405/222	3.06 (2.65–3.54)
By Race		
White		
No	4903/3432	1.00 (ref.)
Yes	822/480	2.60 (2.36–2.86)
Black		
No	780/518	1.00 (ref.)
Yes	136/84	3.24 (2.55–4.12)
Asian/Pacific Islander		
No	553/329	1.00 (ref.)
Yes	61/31	3.36 (2.27–4.96)
Other		
No	223/125	1.00 (ref.)
Yes	17/11	10.40 (4.99–21.68)
By tumor stage		
Stage I		
No	1580/553	1.00 (ref.)
Yes	349/110	3.44 (2.79–4.26)
Stage II		
No	440/246	1.00 (ref.)
Yes	87/40	3.29 (2.32–4.67)
Stage III		
No	1620/1238	1.00 (ref.)
Yes	263/178	2.43 (2.06–2.85)
Stage IV		
No	2509/2181	1.00 (ref.)
Yes	307/257	2.71 (2.38–3.10)
Unknown stage		
No	310/186	1.00 (ref.)
Yes	30/21	1.98 (1.22–3.21)

^1^ HRs were estimated from multivariable time-dependent Cox proportional hazard models. HRs were adjusted for age (as continuous variable), sex, race, year of diagnosis, tumor stage, surgery, chemotherapy, and radiation except the stratified variable itself. HR = hazard ratio; CI = confidence Interval.

## Data Availability

Data for this study came from the Department of Defense and cannot be shared publicly based on the approved IRB protocol and data-sharing agreements.

## Abbreviations

AJCC	American Joint Committee on Cancer
CCR	Central Cancer Registry
CI	Confidence Intervals
COPD	Chronic Obstructive Pulmonary Disease
CPT	Current Procedural Terminology
CT	Computed Tomography
HR	Hazard Ratio
ICD	International Statistical Classification of Diseases and Related Health Problems
LC	Lung Cancer
MDR	Military Health System Data Repository
MHS	Military Health System
NSCLC	Non-Small Cell Lung Cancer
PH	Pulmonary Hypertension
